# European bioeconomy strategies could better integrate sustainability agendas

**DOI:** 10.1007/s11625-025-01752-1

**Published:** 2025-10-13

**Authors:** Anne Warchold, Jing Li, Prajal Pradhan

**Affiliations:** 1 Integrated Research on Energy, Environment and Society (IREES), Energy and Sustainability Research Institute Groningen (ESRIG), University of Groningen, 9747 AG Groningen, The Netherlands; 2 Potsdam Institute for Climate Impact Research (PIK), Member of the Leibniz Association, P. O. Box 60 12 03, Potsdam, Germany

**Keywords:** Sustainability agenda, SDGs, Bioeconomy, Development policy, Governance, Synergies and trade-offs

## Abstract

The bioeconomy is increasingly seen as a key national development strategy for sustainable economic growth, environmental restoration, and achieving the Sustainable Development Goals (SDGs). Numerous countries have introduced strategies to outline their bioeconomy visions, aiming to transition from fossil fuel-dependent systems to bioeconomies that address pressing environmental and socio-economic challenges. Despite this potential, the bioeconomy is not inherently sustainable nor circular, and policymakers pursue bioeconomies to different extents, facing challenges in balancing synergies and trade-offs with the SDGs. This study examined bioeconomy strategies presented in 29 policy documents from 14 European countries and the European Union. Using text analyzing techniques, we evaluate the extent to which bioeconomy strategies determine the salience of sustainability. Our analysis shows that most bioeconomy strategies emphasize opportunities aligned with SDGs 6 and 12–15, focusing on environmental objectives within the planet pillar of the 2030 Agenda. However, these strategies often neglect the principles of “equitable” and “fair & inclusive” transition and fail to consider potential unintended consequences or trade-offs for sustainability. This disconnection is further reflected by a significant divide between evidence on bioeconomy–SDG interactions and the narratives presented in policy documents. The concepts of sustainability and circularity are theoretically integrated into bioeconomy strategies but often remain lethargic, focusing on narratives rather than evidence on existing trade-offs at the expense of human needs and ecosystems. To prevent the risk of unsustainable transitions, bioeconomy strategies should evolve toward systemic approaches that emphasize resilience, equity, and ecological regeneration. Future research should explore practical mechanisms for aligning bioeconomy development with global sustainability goals.

**Supplementary Information:**

The online version contains supplementary material available at 10.1007/s11625-025-01752-1.

## Introduction

The bioeconomy (BE) concept dates back more than 20 years, with a shifted focus from a biotechnology-centered paradigm to a bioeconomy as a transformative force for sustainable development (Patermann and Aguilar 2018). However, diverse perspectives on the concept of bioeconomy have evolved since its emergence in the 1990s (Giampietro 2019), leading to a need for a more integrated approach to its holistic understanding. To address this need, Faulkner et al. (2024a) defines bioeconomy as a concept that rests on four pillars: environmental, economic, social, and governance. These pillars are aligned with the *Aspirational Principles and Criteria for a Sustainable Bioeconomy* agreed on by the United Nations Food and Agriculture Organization (FAO) and the International Sustainable Bioeconomy Working Group (ISBWG) (Bracco et al. 2019). These principles and their criteria provide a framework for a sustainable bioeconomy. Yet, a successful implementation of bioeconomy requires the availability of appropriate policy strategies and governance mechanisms (Filipe 2024). Given the inherent complexity and multisectoral nature of the bioeconomy, effective strategies will require a multi-level framework that leverages synergistic aspects from the four pillars (Ahenkora 2025) and coordination from local to global scales (Filipe 2024).

To make a wider sustainability contribution, bioeconomy strategies need to strongly align with global sustainability frameworks such as the Sustainable Development Goals (SDGs) (Schütte 2018). This is crucial, because no country is on track to achieve all the SDGs by 2030 (Sachs et al. 2024). Promoting bioeconomy could contribute to achieving several SDGs, e.g., food security (SDG 2), bio-based energy (SDG 7), economic growth (SDG 8), and responsible consumption (SDG 12) (Hetemäki et al. 2017; Heimann 2019; Ronzon and Sanjuán 2020; Calicioglu and Bogdanski 2021). However, bioeconomy practices are neither inherently circular nor sustainable (Heimann 2019). For example, promoting bioeconomy for decarbonization (SDG 13) without careful consideration may lead to excessive land (SDG 15) and water use (SDG 6) (Miranda et al. 2023). Thus, reflecting on SDGs holistically in the national bioeconomy strategies is crucial to avoid unintended negative consequences.

Europe is a leading region for bioeconomy research, contributing to most of the related publications (80%) (Ferraz and Pyka 2023). In 2012, the European Commission launched the first-ever European Bioeconomy Strategy to address the rising societal and environmental challenges (European Commission 2012). It aimed to facilitate the transition from a fossil-based society by utilizing biological resources and innovative technologies. This strategy has sparked discourse, research, and innovation across various sectors and countries. Bioeconomy research within the Programme Horizon 2020 (2014–2020) and the creation of a public-private partnership of bio-based industries impacted the concept further (Patermann and Aguilar 2018), initiating a review process of its initial strategy.

In 2018, the European Commission updated its bioeconomy strategy and action plan (European Commission 2018a, b). It introduced a sustainable European bioeconomy, contributing to the achievement of the SDGs and the fulfillment of the Paris Agreement. The updated strategy and action plan expanded the five targets from the 2012 strategy (i.e., food security, sustainable natural resource management, increasing bioresources, climate change mitigation and adaptation, and job creation) with three action areas. They are (i) scaling up bio-based sectors and markets, (ii) deploying bioeconomies across Europe, and (iii) dealing with the ecological boundaries of the bioeconomy. By defining the bioeconomy as a pathway to sustainable economic growth and achieving SDGs, the 2018 strategy prompted many countries to adopt national strategies to guide policies and investments. These national strategies adopted the proposed definition, aligning with the EU strategy but emphasizing areas of particular importance to their economy (Gardossi et al. 2023). These strategies depict the countries’ commitment to leveraging the bioeconomy for sustainable transformation. However, limited studies reflect their alignment with the wider sustainability frameworks, e.g., SDGs and bioeconomy principles.

Previous studies have reviewed scientific publications (Bugge et al. 2016; D’Amato et al. 2017; Holmgren et al. 2020), deciphered media discourse (Sanz-Hernández et al. 2020), investigated people’s perspectives (Eversberg and Fritz 2022), or discussed stakeholder narratives (Vivien et al. 2019) on bioeconomy. Virgolino and Holden (2025) assessed the extent to which bioeconomy literature aligns with the FAO’s ten principles for a sustainable bioeconomy, highlighting major blind spots in social and environmental dimensions. Beyond these discursive and societal perspectives, another body of work has examined the policy dimension of the bioeconomy. Some studies combined document analysis and expert interviews to examine how competing actors negotiate biophysical limits in EU biomass regulation conflicts (Fleischmann et al. 2024), or to investigate the alignment of European-funded research and innovation projects with bioeconomy policy priorities (Brandão and Santos 2024). Others have explored bioeconomy’s typologies using a mix of literature, selective policies documents, and/or surveys in specific regions, such as the Baltic Sea (Vanhamäki et al. 2019), using regional case studies (Skondras et al. 2024), in specific countries, such as Germany (Bogner and Dahlke 2022), in Europe (Hausknost et al. 2017; De Besi and McCormick 2015), or using worldwide samples (Proestou et al. 2024). For instance, Dietz et al. (2018) examined the objectives and instruments of 41 national bioeconomy-related policies, focusing on their sustainability dimensions in a broader conceptual sense. Similarly, Maksymiv et al. (2021) reviewed strategic challenges of bioeconomy governance in relation to a subset of SDGs (2, 3, 7, 9, 12–15). These contributions underline the importance of linking bioeconomy strategies to sustainability frameworks, yet they vary in scope and focus, ranging from subsets of SDGs to regional cases or literature analyses, without systematically covering European strategies in their entirety.

To address these gaps, this paper systematically analyzes bioeconomy strategies across European countries, at the regional level, and the EU, reflecting on them in relation to wider sustainability frameworks. While previous work, such as Dietz et al. (2018), examined the objectives and instruments of bioeconomy-related policies across multiple countries, our study advances this line of research in several ways. First, we systematically assess the alignment of strategies with the comprehensive FAO’s ten principles for a sustainable bioeconomy, which has not yet been applied to policy strategies. Second, we complement this with an evaluation against the entire framework of the 17 SDGs and 169 targets, whereas earlier studies primarily focused on broader sustainability dimensions or selected SDGs. Third, we incorporate strategies from the EU, national, and regional levels, enabling a cross-country and cross-scale comparison that has been largely absent from prior research. In particular, we examined opportunities and challenges for accelerating progress on the SDGs, in regard to, e.g., climate action, sustainable production and consumption, and biodiversity conservation. By doing so, the paper contributes to ongoing debates on the role of the bioeconomy in achieving sustainable development in Europe and beyond, offering a comprehensive overview of current strategies’ visions and their implications for sustainability.

## Materials and methods

Our research method comprises two complementary steps. First, we performed a text analysis of bioeconomy strategies to map their alignment with two sustainability frameworks, i.e., bioeconomy principles and SDGs. Second, we compared the identified political foci to evidence of ex-post analysis of SDG and bioeconomy interactions.

### Bioeconomy strategies

Our analysis focuses on official government documents, available in English, retrieved from the European Commission’s regularly updated database (European Commission 2024). When multiple national strategies were available, we only included the most recently published one. As of September 2024, national bioeconomy strategies and action plans were available for eleven European countries, namely Austria, Estonia, Finland, France, Germany, Ireland, Italy, Latvia, Norway, Spain, and The Netherlands (Table 1 and Fig. S1). Belgium, France, Germany, Latvia, Poland, and Romania also published regional strategies. Together with the four European-centered, we collected the full text in PDFs of 29 bioeconomy strategies, including national and regional strategies. However, we did not include other documents related to the bioeconomy (e.g., circular economy strategies) or macro-regional (e.g., the Nordic bioeconomy strategy) bioeconomy strategies to focus our study on bioeconomy documents at the European, national, or regional level.

**Table 1:** Overview of European bioeconomy (BE) strategies, including details on their scale, type, publication year, and references to the 2030 Agenda and its Sustainable Development Goals (SDGs) introduced in 2015

Level	Bioeconomy strategy type	Region	Publication year	2030 Agenda mentioned
EU	Strategy launch		2012	–
	Strategy update		2018	Yes
	Action plan		2018	Yes
	Progress report		2022	Yes
Austria	National strategy		2019	Yes
	BE flagship projects		2021	Yes
Belgium	Regional strategy	Flanders	2020	No
Estonia	National strategy		2023	No
Finland	National strategy		2022	Yes
France	National strategy		2017	No
	National action plan		2018	No
	Regional strategy	Pays de la Loir	2020	No
Germany	National strategy		2020	Yes
	National action plan		2020	Yes
	Regional strategy	Baden-Württemberg	2019	No
	Regional strategy	Bayern	2020	Yes
	Regional strategy	Metropolitan area Frankfurt Rhine Main	2021	No
Ireland	National strategy		2018	No
	National action plan		2023	No
Italy	National strategy		2019	Yes
	National action plan		2021	Yes
Latvia	National strategy		2017	No
	Regional strategy	Vidzeme	2020	No
Netherlands	National strategy		2018	No
Norway	National strategy		2016	No
Poland	Regional strategy	Mazowieckie Voivodeship	2022	No
Romania	Regional strategy	Sud Muntenia	2019	No
Spain	National strategy		2016	No
	Regional strategy	Catalonia	2021	Yes

### Sustainability frameworks

**Fig. 1:**
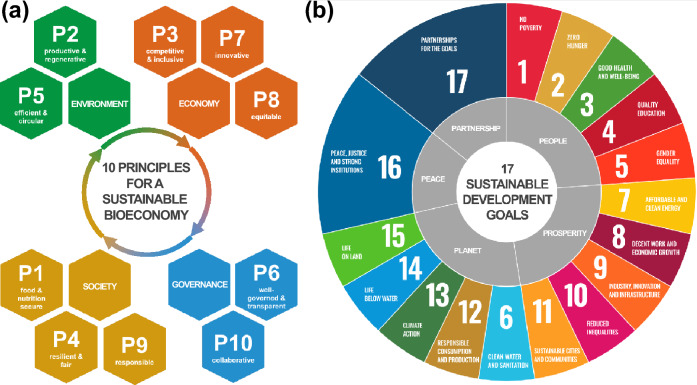
Bioeconomy framework and the 2030 Agenda for Sustainable Development: The panels illustrate the 10 principles (P) for a sustainable bioeconomy (BE) [(**a**), (FAO 2021)] alongside the 17 Sustainable Development Goals (SDGs) of the 2030 Agenda (**b**). The BE principles are categorized according to the sustainability dimensions: environment, economy, society, and governance, and the holistic and integrated vision of sustainable BE. Detailed descriptions of BE principles and criteria are listed in Table S1. Similarly, the SDGs are structured around the five pillars of the 2030 Agenda: people, prosperity, planet, peace, and partnership. In the SDG wheel, Goals 16 and 17 are given greater visual space to highlight their enabling role for progress across all other goals, underscoring the interconnected nature of the 2030 Agenda and the need for balanced, mutually reinforcing progress across pillars

The transition toward a bioeconomy poses complex and multifaceted challenges, with synergies and trade-offs (Warchold and Pradhan 2025). As bioeconomy practices might not be inherently circular or sustainable (Heimann 2019), establishing bioeconomy strategies that encourage the integration of incentives for sustainable innovation and aligning them with sustainability goals is crucial. Therefore, we applied two sustainability frameworks to identify commonalities and differences among the bioeconomy strategies (Fig. 1). Doing so enables a comparative analysis of how these sustainability frameworks are integrated into the strategies. The two frameworks are the *Aspirational Principles and Criteria for a Sustainable Bioeconomy* (Bracco et al. 2019) and the *2030 Agenda for Sustainable Development* adopted by the United Nations General Assembly (UN. General Assembly 2015).

FAO and ISBWG agreed on a set of ten principles, 24 criteria, and diverse indicators, offering a comprehensive tool for monitoring the transition toward a sustainable, circular bioeconomy (Bracco et al. 2019) [Fig. 1a; full description in Table S1]. This bioeconomy framework covers social, economic, environmental, and governance aspects of sustainability, ensuring that the bioeconomy not only meets the needs of the present but also safeguards the well-being of future generations (FAO 2021). For example, in construction, wood offers environmental benefits as well as economic opportunities. Using engineered wood for urban construction could save up to 106 gigatonnes (Gt) of greenhouse gas emissions by 2100 while meeting demand through expanded timber plantations without significantly impacting agriculture (Mishra et al. 2022). Moreover, the circular economy is central to the bioeconomy, focusing on minimizing waste and maximizing resource efficiency by closing production and consumption loops (Mesa et al. 2024). Also, anaerobic digestion contributes to circularity by transforming organic waste into renewable biogas and useful byproducts, reducing waste and creating value from discarded materials (European Commission 2018a).

In the bioeconomy principles, the circularity aspect is reflected by incorporating criteria on resource efficiency in production and consumption, waste management, the extended lifecycle of materials, and fostering competitiveness and innovation (Bracco et al. 2019). The conceptual boundaries, however, remain fluid, multifaceted, complementary and intersecting (Stegmann et al. 2020; Bugge et al. 2016; De Besi and McCormick 2015; Rodriguez-Anton et al. 2019).

The 2030 Agenda as a blueprint for sustainable development, consisting of 17 SDGs and 169 targets, was adopted in 2015 (UN. General Assembly 2015). At the heart are five critical dimensions encapsulating sustainability: people, prosperity, planet, partnership, and peace (Fig. 1b). Both frameworks are goal-oriented and have a similar structure. They comprise overarching bioeconomy principles or SDGs, multidimensional criteria or targets, and numerous proposed indicators to monitor progress.

Reflecting bioeconomy strategies based on these two frameworks allows for understanding their contributions to wider sustainability. Therefore, we developed the search query data based on the description of bioeconomy criteria (Data S1). We also adopted the query data on SDG targets from Pradhan et al. (2024) by adding additional synonyms to capture a broader range of occurrences (Data S2). For each bioeconomy criterion and SDG target, these data consist of search queries constructed using keywords, Boolean, and proximity operators and tailored to the Corpus Query Language. One can apply these queries to documents, e.g., PDFs, using the “corpustools” package in the R programming language. In other words, our query data define each of the 24 BE criteria and the 169 SDG targets by a set of keywords.

We use the terms “agenda" and “framework" interchangeably. While agendas define sustainability goals and plans, and frameworks offer tools and methods for assessment and implementation, both entities of our analysis—SDGs and the bioeconomy—function as agendas and frameworks simultaneously.

### Analysis

We adapted the method from Pradhan et al. (2024) to reflect on the two sustainability frameworks in the selected bioeconomy strategies. The procedure combined automated text mining with in-depth manual analysis, similar to previous applications that explored the coverage of SDG targets in IPCC reports (Pradhan et al. 2025). Each BE criterion and SDG target was mapped in the strategies using the *corpustools* package in R, which enabled keyword extraction and corpus linguistics (list of BE and SDG keywords in Data S1 and S2, respectively). Based on the keywords, a database of mapped text segments was compiled for every BE criterion and SDG target across all strategies. We excluded texts in figures, tables, or without sufficient context. Following manual verification, we quantified the frequency of mapped texts per BE criterion and SDG target, and then aggregated the results by respective BE principles and SDGs.

A sentiment analysis followed our text mapping. For each mapped SDG target, we assessed whether the strategy framed it as an opportunity or a challenge. The sentiment analysis was performed only for the mapped SDG targets, because our aim was to assess how the bioeconomy is portrayed as affecting SDG achievement. Using the *tidytext* package in R, which applies predefined sentiment lexicons, we first identified those sentiment-bearing words for each mapped segment for each SDG target. Typical positive sentiment words included, e.g.,“advance”, “support”, “achieve”, or “protect”, while negative sentiment words included, e.g.,“risk”, “threat”, “damage”, or “decline”. Following Pradhan et al. (2024), ambiguous terms, such as “sustainable”, “sustainability”, “poor”, “poverty”, and “waste”, were excluded, as they often occur in BE and SDG definitions but do not inherently indicate sentiments. Positive associations were classified as opportunities, while negative ones as challenges. All automated results were subsequently verified manually in their original context, ensuring that the sentiments reflected the actual framing of the strategy rather than relying solely on automated word counts. Afterward, we manually assessed the mapped text and synthesized opportunities and challenges related to each SDG target, which are reflected in the strategies (Data S3).

Finally, we compared the opportunities and challenges identified in the strategies with existing evidence on synergies and trade-offs between the bioeconomy and the SDGs published by Warchold and Pradhan (2025), The authors investigated SDG–bioeconomy interactions for European countries based on empirical data using the FAO’s bioeconomy framework and a unified SDG database. Here, we explicitly distinguish between the two concepts: synergies and trade-offs are evidence-based interactions observed ex-post using empirical analyses, whereas opportunities and challenges denote how strategies frame potential contributions or risks, typically with a forward-looking orientation. In some cases, these opportunities and challenges are grounded in existing evidence, while in others they remain aspirational or indicative of future intentions. Opportunities may translate into synergies, and challenges into trade-offs, but such linkages remain conditional. To examine this joint structure of strategy framings and ex-post evidence, we applied a weighted principal component analysis (PCA) to the relative shares of opportunities, challenges, synergies, and trade-offs associated with each SDG. To avoid overemphasizing rarely mentioned SDGs that nonetheless showed extreme percentages (e.g., 100% opportunities with very few statements), we weighted the percentages by the log of the number of strategy references. The PCA thus captures both the balance of positive and negative framings and the relative coverage of each SDG in the strategies, while reducing the influence of low-frequency cases. Thus, the main patterns of variation are visualized, and the relative positioning of SDGs is directly compared.

## Results

All bioeconomy principles and SDGs are integrated into the EU, national, and regional bioeconomy strategies. They, however, differ in the extent and level of provided details on the different bioeconomy criteria and SDG targets. This reflects that the strategies address a broad range of sustainability goals but focus more specifically on areas of particular relevance to the bioeconomy. The majority of the strategies fail to consider potential unintended consequences for sustainability, nor do they identify clear impacts toward the SDGs.

### Integration of bioeconomy principles in development strategies

**Fig. 2:**
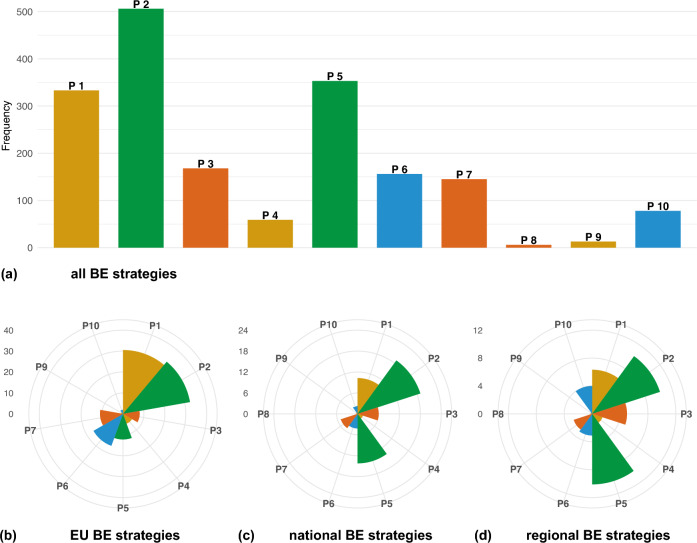
Frequency of bioeconomy (BE) principles mapped summed up for all documents (**a**) in the European Union (EU) (**b**), national (**c**), and regional (**d**) BE strategies. The environmental dimension—Principles 2 and 5—is the primary focus at the EU, national, and regional levels. Detailed descriptions of BE principles and criteria are listed in Table S1. To enable comparison across scales, SDG occurrence values for EU, national, and regional strategies are normalized by the number of documents associated with each scale (EU = 4, national = 15, and regional = 9)

Bioeconomy principles emphasizing food security (Principle 1), efficient use of natural resources (Principle 5), their protection, and climate change mitigation (Principle 2) are central to all the bioeconomy strategies. While we mapped all ten bioeconomy principles and 24 criteria (Table S1) in the strategies, their integration varies (Fig. 2). We categorized the mapping into three levels based on the frequency: strong integration of Principles 1 (food & nutrition secure), 2 (productive & regenerative), and 5 (efficient & circular), with 333–506 references; moderate integration of Principles 3 (competitive & inclusive), 6 (well governed & transparent), and 7 (innovative), with 145 to 168 references; and weak integration of Principles 4 (resilient & fair), 8 (equitable), 9 (responsible), and 10 (collaborative), with 6–78 references (Fig. 2a).

Comparing bioeconomy principle integration across scales, we find that the environmental dimension—Principles 2 and 5—is the primary focus at the EU, national, and regional levels (Fig. 2b–d). However, while Principle 5 is more prominent at the regional level, its importance decreases at the EU level, whereas Principle 10 gains significance moving from the EU to national and regional scales. The updated EU Bioeconomy Strategy and Action Plan emphasizes inclusiveness, recognizing that progress depends on collaboration among all stakeholders. However, at the national level, such efforts (Principle 10) are notably less pronounced. Regional strategies, such as Romania’s, place a strong emphasis on collaboration (Criterion 10.1), particularly in need of technology transfer.

**Fig. 3:**
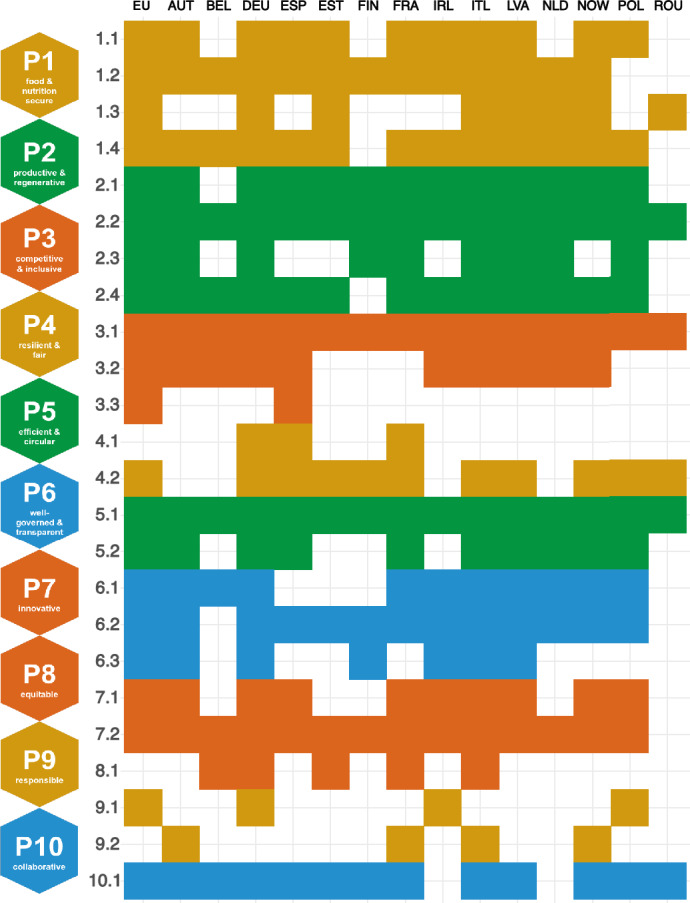
Bioeconomy (BE) criteria mapped across the bioeconomy strategies. Criteria 2.2 and 5.1 are referenced in the bioeconomy strategies of all countries and the European Union (EU), while the coverage of other criteria varies. Country names are abbreviated according to UN country coding. Detailed descriptions of BE principles and criteria are listed in Table S1. The coloring is according to the BE color coding of principles; see icons on the left

Criteria 2.2 and 5.1 are referenced in the bioeconomy strategies of all countries and the EU (Fig. 3). Criterion 2.2 is frequently highlighted by keywords related to “reducing emissions” and “climate change adaptation”. Criterion 5.1 focuses on improving resource efficiency and biomass use, with frequent mentions of “efficient resource use” and “recycling waste”. Strategies with a strong focus on Criterion 5.1, like Germany or Italy, often strongly link to reducing food waste and loss (Criterion 5.2), and aligning their potential with Principle 1, such as organic farming (Criterion 1.4). Both Germany and Italy rank among the top three waste-generating nations in the EU in 2022, producing approximately 385 and 190 million metric tons of total waste, respectively (European Commission, Eurostat 2024). The bioeconomy can partially contribute to waste reduction by promoting circular value chains, valorizing residues, and utilizing biomass in a cascading manner (Pinheiro and Symochko 2025). In this context, Finland, for example, is also developing innovative bio-based alternatives to plastic packaging using wood fibers, illustrating how national resources shape strategic priorities. Finland emphasizes a forest-based bioeconomy, with about 72% of its land area under forest cover and forest products accounting for more than 70% of bioeconomy export value (Finnish Government 2022).

Another principle that is often mentioned, but less frequently, is Principle 7, which emphasizes the importance of using and generating knowledge, innovation, and sound technology. Beyond packaging and agriculture, bio-based solutions are revolutionizing medicine and construction, creating innovation for circularity. Bioimpression technologies are printing tissues and organs, potentially transforming organ transplants, and reducing dependence on donor organs (Murphy and Atala 2014). This exemplifies how bioeconomic principles can address critical societal needs while promoting sustainability. In construction, bio-based materials like mycelium (fungus) and hempcrete (hemp-based concrete) offer sustainable alternatives to traditional building materials (Alaneme et al. 2023; Yang et al. 2024). These materials are biodegradable and provide improved insulation properties, reducing the energy demand for heating and cooling buildings.

Criteria 3.3 (resilience of rural and urban economies), 4.1 (sustainability of urban centers), and 8.1 (supporting local economies through biomass trade) all highlight the local context of the bioeconomy. However, these aspects are not strongly emphasized in bioeconomy strategies, even at the regional level, where local dynamics could play a significant role in shaping bioeconomy initiatives.

### Integration of SDGs in bioeconomy strategies

We mapped all 17 SDGs and 70 of 169 targets in the bioeconomy strategies. This indicates that bioeconomy strategies address a broad range of sustainability goals, but focus more specifically on areas of particular relevance to the bioeconomy. Nonetheless, only half of the strategies explicitly reference the 2030 Agenda or the SDGs (Table 1). However, conceptually, all goals and slightly less than half of the targets are integrated into these strategies.

**Fig. 4:**
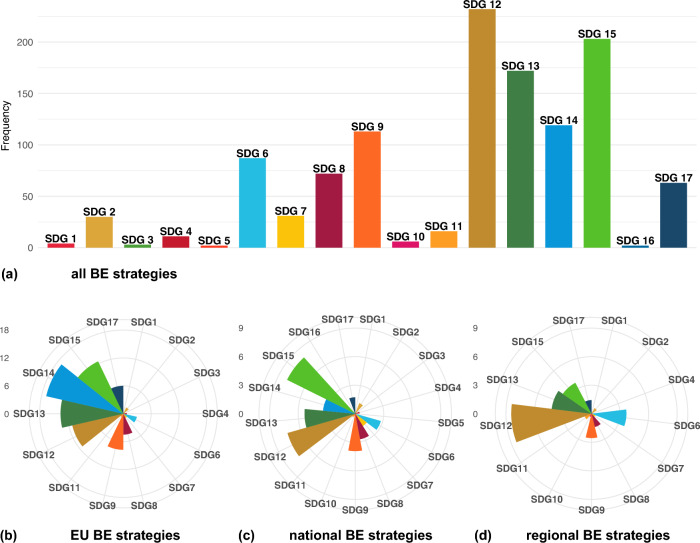
Frequency of Sustainable Development Goals (SDGs) mapped for all (**a**), the European Union (EU) (**b**), national (**c**), and regional (**d**) bioeconomy (BE) strategies. To enable comparison across scales, SDG occurrence values for EU, national, and regional strategies are normalized by the number of documents associated with each scale (EU = 4, national = 15, and regional = 9)

SDGs 12 (Responsible consumption and production), 13 (Climate change), and 15 (Life on land) are the top three goals mapped (Fig. 4a). The top three mapped targets belong to the same SDGs, namely SDG 12.3 (reduce food waste and losses), 13.1 (enhance climate resilience), and 15.1 (promote ecosystem conservation, restoration, and sustainable use). Target 13.1 is the only target all countries and the EU referenced in their bioeconomy strategies. The least mapped goals are SDG 3 (good health and well-being), 5 (gender equality), and 16 (peace, justice and strong institutions), which are mentioned in fewer than three strategies.

Comparing SDG integration across the strategies, we find that the planet pillar—SDGs 6 and 12 to 15—is the primary focus at the EU, national, and regional levels (Fig. 4b–d). While SDG 14 is prioritized at the EU level, its significance declines regionally, in contrast to SDGs 6 and 12, which grow in importance from regional to national to EU scales. No regional strategy mentions SDG 14, despite some regions having coastal borders (Fig. S1). Yet, we observe a strong emphasis in the Norwegian strategy on sustainable consumption (SDG 12) and marine resources (SDG 14). This focus reflects Norway’s natural endowments as a major fisheries nation, where seafood constitutes a central component of the bioeconomy (Norwegian Ministry of Trade, Industry and Fisheries 2016). While this creates opportunities, it also entails trade-offs, as expansion of aquaculture and seafood production raises concerns over environmental impacts, spatial conflicts, and balancing economic growth with ecosystem sustainability (Pleym et al. 2021).

The prosperity pillar (SDGs 7–11) and the partnership pillar (SDG 17) show relatively even focus across all scales. For example, Germany’s bioeconomy strategy shows strong integration of SDG 8 and 9, reflecting its wood- and industrial-based orientation. The strategy is characterized by a techno-economic focus that prioritizes industrial innovation, competitiveness, and resource efficiency (Bogner and Dahlke 2022), while also maintaining a broader bioresource-oriented perspective that mobilizes biomass to integrate into industrial processes with a circularity objective (Gottinger and Proestou 2025). In contrast, the people pillar is unevenly prioritized and almost absent at the regional scale. Meanwhile, the peace pillar (SDG 16) is referenced only in the Norwegian strategy concerning effective institutions (target 16.6).

SDG 2 (zero hunger) is the goal most mentioned in the people pillar, in eight countries and the EU, most referring to SDG target 2.3 (double productivity and income of small-scale food producers) and 2.a (invest in rural infrastructure, agricultural research, technology, and gene banks). Latvia, for instance, outlines concrete action plans to support small-scale producers, particularly farms and family-based enterprises (Target 2.3), in its national and regional bioeconomy strategies. This aligns with the EU’s 2018 strategy, which calls for mechanisms to support a diverse agri-food supply chain, including a multitude of small-scale, family-based producers, retailers, and food service outlets that operate alongside larger, globalized companies. Also, we observe that Italy places a strong focus on food systems, addressing food supply chains, prices, diets, and security, as the only country referencing SDG targets 2.1, 2.3, 2.4, 2.a, and 2.c. For instance, Italy emphasizes alternative food sources, such as insects and algae, and novel food microbes, utilizing pedoclimatic national areas, encouraging short local food chains to enhance health while countering non-market food networks (Italian Government 2019). Additionally, Italy emphasizes encouraging youth and women to engage in farming and other rural, coastal, and marine activities (NBCD 2021). Furthermore, Italy references all observed SDG 14 targets (14.1–14.5) and SDG 15 targets (15.1, 15.2, 15.3, 15.5, 15.6, 15.8).

**Fig. 5:**
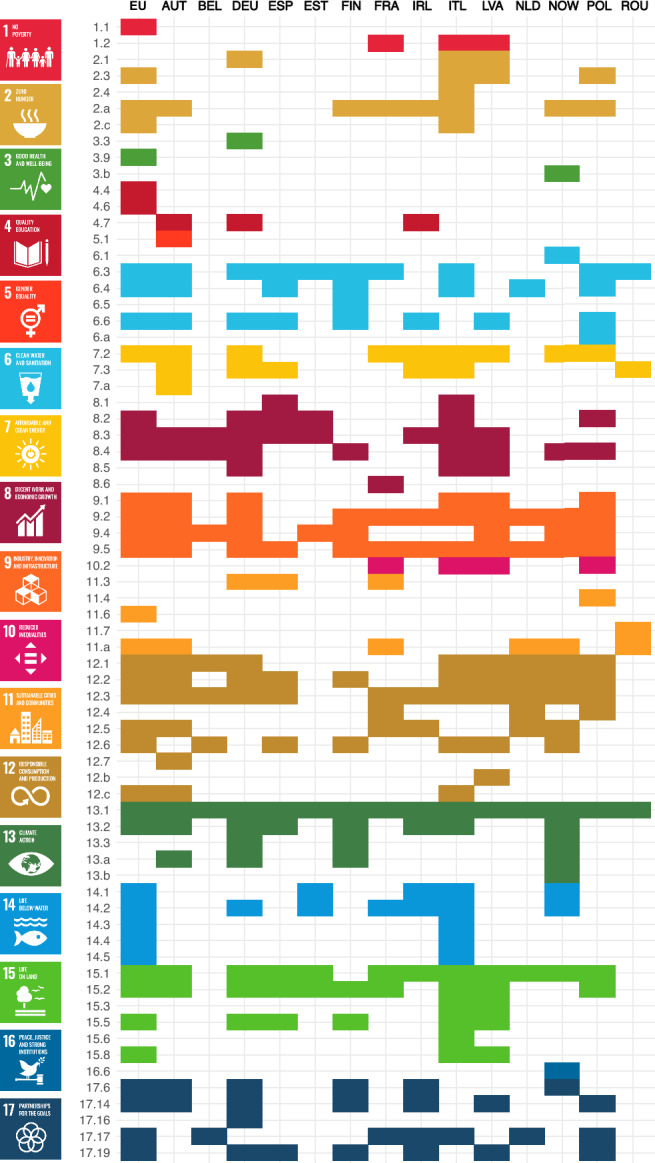
Sustainable Development Goal (SDG) targets mapped across the bioeconomy strategies for the European Union (EU) and countries. Country names are abbreviated according to UN country coding. The coloring is according to the UN color coding of SDGs; see icons on the left

Comparing the SDG coverage of the 2012 and 2018 EU bioeconomy strategies, we observe a shift in focus and extent (Fig. S2). Although the 2012 strategy was published before the launch of the 2030 Agenda, the EU had already addressed a wide range of topics related to the SDGs. SDGs 9, 12, and 13 are strongly integrated, while SDGs 8 and 17 are moderately considered (Fig. 5). The main emphasis is, therefore, to enhance the EU economy, providing new employment and business possibilities. In contrast, the aspects of sustainability and resource availability are addressed only to a limited extent. The focus shifted in the 2018 agenda toward a more environmentally driven perspective, with SDGs 14 and 15 becoming the most prominently addressed goals. Overall, the frequency of SDG targets mentioned increased fivefold.

We observed some regional strategies that replicated objectives from the 2018 EU bioeconomy strategy. For example, the Mazovian bioeconomy strategy emphasizes accelerating the transformation to healthy, nutrition-sensitive, resource-efficient, resilient, circular, and inclusive food and farming systems as one of its major objectives. This means that for some principles and goals, regional plans are closely aligned with EU objectives, reflecting a top–down influence and ensuring consistency with broader EU goals while adapting them to local contexts.

### Opportunities and challenges for SDG acceleration in bioeconomy strategies

**Fig. 6:**
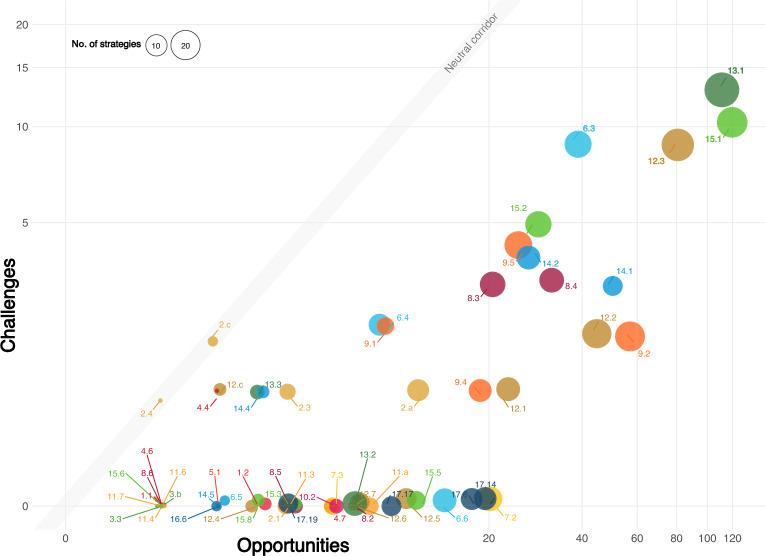
Bioeconomy opportunities and challenges in development strategies mapped in relation to Sustainable Development Goal (SDG) targets. Each point represents an SDG target, colored by its respective goal. The size reflects the number of bioeconomy strategies referencing the target, while scaling indicates the frequency of these references across strategies

We observe uneven positive and negative sentiments in the mapped texts associated with SDGs in bioeconomy strategies. Among the 70 mapped SDG targets, sentiments were identified for 60, revealing distinct clusters (Fig. 6). Data S3 provides a summary of sentiments regarding opportunities and challenges in the bioeconomy in relation to SDG targets.

SDG targets related to the planet pillar (SDGs 6, 12–15) and economic growth and industrialization (SDGs 8, 9) are the most frequently referenced. However, the challenges are comparatively marginal, as these targets remain in the opportunity corridor, indicating a predominantly positive framing within bioeconomy strategies. For example, SDG target 13.1 (climate resilience and adaptive capacity) is mentioned as an opportunity 109 times across all 29 strategies, with only 13 references to challenges. This strong emphasis reflects the central role of climate change mitigation in European policy, aligning closely with the narratives of the Paris Agreement and the European Green Deal that prioritize reducing carbon emissions through bio-based solutions. Thus, opportunities are primarily centered on leveraging biomass, sustainable farming, and bio-based materials to reduce emissions and enhance resilience (European Commission. Directorate General for Research and Innovation 2012; BMBF and BMEL 2020). Challenges, by contrast, relate to the high costs of carbon capture infrastructure, persistent agricultural emissions, and the need to align productivity gains with both environmental sustainability and animal welfare (European Commission. Directorate General for Research and Innovation 2012, 2018; BMNT et al. 2019) (Data S3).

36 SDG targets, such as 6.6 (water-related ecosystems), 7.2 (renewable energy share), 17.14 (policy coherence), and 17.16 (global partnership), form an opportunity-only cluster, as being exclusively linked to positive sentiments, with no challenges mentioned. For instance, while SDG 7 targets are comparably moderately referenced, yet when they are mentioned, they are presented solely as opportunities. This limited but positive framing contrasts with empirical research. While biomass can contribute to cost-effective climate mitigation, its large-scale deployment may also create challenges, such as resource competition, land-use pressures, higher system costs, and dependence on carbon capture (Warchold and Pradhan 2025; Millinger et al. 2025). Taken together, this suggests that current strategies may underestimate both the potential and the risks of bio-based energy within the broader European renewable energy context.

SDG targets 2.4 (sustainable food production and resilient agriculture) and 2.c (food commodity markets and trade restrictions) are exceptions to those patterns, which are referenced with equal positive and negative sentiment. The EU and Italy, for instance, highlight both the opportunities and challenges of bioeconomy in the agricultural sector, particularly regarding price volatility, and that increasing the value added by the bioeconomy food and fish sectors should not be done by importing from countries with less strict environmental regulations (European Commission. Directorate General for Research and Innovation 2012; Italian Government 2019).

The EU bioeconomy strategies highlight the opportunities with evidence-based statements. For example, studies on wood use in buildings consistently show that wood products generate lower greenhouse gas emissions than alternative materials over their entire life cycle, including use and disposal (Himes and Busby 2020; Migoni Alejandre et al. 2024). Meta-analyses suggest that replacing concrete with wood can lead to an average reduction of 2.1 tons of carbon dioxide emissions per 1 ton of wood products (Hurmekoski 2017). Other strategies often present opportunities as direct effects without evidence, semantically akin to claims like “The bioeconomy significantly contributes to reducing greenhouse gas emissions and halting biodiversity loss.", without substantiating their validity. Such statements contribute to the significant positive associations with targets belonging to SDGs 13 and 15. In these bioeconomy strategies, challenges in the bioeconomy transition are often framed around current limitations, such as insufficient innovation or investments. If mentioned, they are not always directly linked to the transition itself but often to broader sustainability challenges. For example, in the context of SDG target 12.3, Italy highlights the “social reluctance to change dietary behavior and reduce food-waste generation” (Italian Government 2019). Also, less attention is given to potential future trade-offs, such as increased biomass demand competing for resources like arable land and water, which could displace food crops and drive up food prices. Italy is among the few countries dedicating several pages to sector-specific opportunities and challenges in agriculture, forestry, marine bioeconomy, food, and bio-based industries. The strategy outlines targeted actions to address these issues.

### Alignment of bioeconomy strategies with evidence on SDG–bioeconomy interactions

**Fig. 7:**
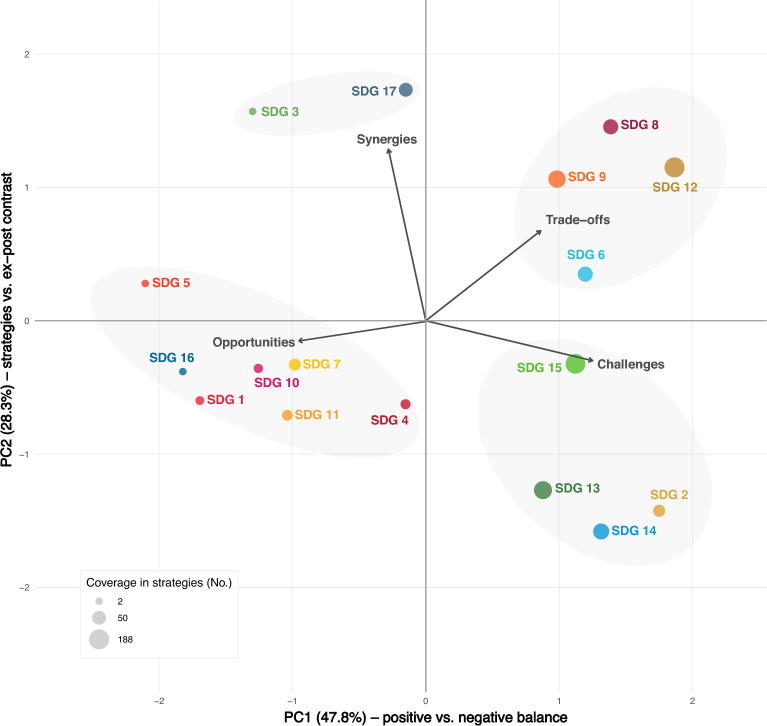
Bioeconomy (BE) opportunities and challenges in achieving the Sustainable Development Goals (SDGs) contrasted with ex-post evidence of synergies and trade-offs between BE–SDG interactions by Warchold and Pradhan (2025). Coverage in the strategies is shown based on statements with positive and negative sentiments mapped to the SDGs, with bubble size reflecting the number of strategy references. PCA was applied to reduce the four dimensions (opportunities, challenges, synergies, and trade-offs) into two principal components that capture the dominant variation between optimistic policy framings and empirically observed interactions. Four clusters of SDGs were identified in this PCA space using k-means clustering ($k = 4$). Arrows indicate the orientation of the four variables in the PCA space

Our analysis reveals a significant discrepancy between bioeconomy strategies and ex-post evaluations of the existing SDG–bioeconomy interactions. While strategies predominantly emphasize opportunities and synergies, assessments highlight notable trade-offs and negative impacts, particularly in specific SDGs (Fig. S3). Social and institutional SDGs are predominantly framed as opportunities in bioeconomy strategies, whereas environmental goals are more strongly linked to trade-offs and mixed outcomes, forming distinct clusters that underline the contrast between policy framings and observed impacts (Fig. 7).

Environmental SDGs are addressed in strategies with a relatively balanced mix of opportunities and challenges, reflecting a recognition of both positive and negative impacts. Economic SDGs exhibit more substantial evidence of trade-offs, but challenges are less extensively discussed. SDG 2 (zero hunger), SDG 12 (responsible consumption and production), and SDG 14 (life below water) exhibit more than 50% negative impacts from bioeconomy transitions, highlighting challenges, such as increased competition for land, food security risks, and potential harm to marine ecosystems (Muscat et al. 2021; Warchold and Pradhan 2025). The strategies, however, only referred to challenges of at least 10% for SDGs 2, 6 (clean water), and 13 (climate action). The data evaluations indicate a greater prevalence of challenges than strategies initially recognized, reinforcing the need for more comprehensive risk assessments.

SDG 1 (no poverty), SDG 3 (good health), SDG 4 (quality education), SDG 5 (gender equality), SDG 7 (clean energy), SDG 10 (reduced inequalities), SDG 11 (sustainable cities), SDG 16 (peace and justice), and SDG 17 (partnerships)—primarily from the people, peace, and partnership pillars of the 2030 Agenda—are significantly less integrated into bioeconomy strategies. One cluster groups social and institutional SDGs (1, 4, 5, 7, 10, 11, 16), which are characterized by relatively low coverage in the strategies and a framing that is predominantly opportunity-driven, suggesting a more aspirational role for the bioeconomy in these domains. When mentioned, they are almost exclusively framed in a positive light, with little to no discussion of potential trade-offs. This corresponds to the limited consideration in evidence discourses of implication for and by the bioeconomy (Heimann 2019; Ronzon and Sanjuán 2020). SDGs 3 and 17 appear at the periphery of the PCA space, reflecting a more distinct positioning in the bioeconomy discourse. While they are framed positively in strategies, their associations diverge from the other clusters, indicating a more isolated but consistent narrative. Both SDGs are supported by some relative evidence of synergies (Warchold and Pradhan 2025), where, e.g., partnership with indigenous people (Astolfi et al. 2025), firms’ partnership (Ayoub et al. 2024), and strategic collaboration in the bioeconomy is essential (Yang et al. 2024). Similarly, the literature has highlighted links between the bioeconomy and health (Haines 2021; Murphy and Atala 2014; Woźniak and Tyczewska 2021) and recognized them in European Commission documents (European Commission: Directorate-General for Research and Innovation 2015). Yet, this lack of systematic consideration of such goals makes it difficult to assess clear dependencies between the bioeconomy and these broader societal goals, potentially overlooking critical socio-economic implications.

## Discussion

Despite scientific advancements in understanding climate change and sustainability challenges, development strategies and policies—such as the bioeconomy—remain lethargic, often prioritizing positive narratives about opportunities for change while overlooking potential trade-offs. By examining EU, national, and regional bioeconomy strategies, we assessed whether they integrate bioeconomy principles and the 17 SDGs. We evaluated the extent to which these strategies determine the salience of sustainability. Leveraging synergies and tackling trade-offs by understanding the drivers of change behind bioeconomy transitions enables us to meet climate and sustainability goals. To this end, we analyzed opportunities and challenges mentioned within each strategy and compared results to the existing SDG–bioeconomy interactions, bringing several novel findings.

First, in terms of the salience of sustainability, bioeconomy strategies integrate all bioeconomy principles and SDGs, though to varying degrees. Moving beyond this level of overarching aspirations and general alignment, we identified a fragmented coverage of bioeconomy criteria and SDG targets. For example, instead of assessing broad aspirations like “responsible consumption and production” (SDG 12), we could dissect specific elements, such as “efficient use of natural resources” (Target 12.2), “food waste and loss” (Target 12.3), or “sustainable, public procurement” (Target 12.7). By identifying finer nuances within bioeconomy strategies, we can effectively dissect the multidimensional aspects of each principle and SDG. While bioeconomy strategies broadly align with the general principles and goals of the 2030 Agenda, their coverage of the multidimensional aspects remains fragmented. This fragmentation resonates with the wider debate on how to measure and monitor bioeconomy, which, due to its comprehensive scope, is challenging to assess unambiguously (Dolge et al. 2023). Bioeconomy strategies support the political priorities, but no specific EU bioeconomy legislation exists. Since the bioeconomy is intended to stimulate international trade, the absence of an agreed-upon international definition (D’Amato and Korhonen 2021) hampers systematic assessment and international comparability. Utilizing the FAO’s bioeconomy framework and the 2030 Agenda, along with its 17 SDGs as an entry point, allows for comparing countries’ ambitions and pinpoints to key areas for improvement.

Second, we identify similarities and differences across EU, national, and regional bioeconomy strategies. Objectives linked to overarching frameworks such as the Green Deal and the Paris Agreement—addressing climate change adaptation (Principle 2, SDG 13), forest conservation (Principle 2, SDG 15), and circular economy principles (Principle 5, SDG 12)—are consistently prioritized across all levels. At the EU level, agricultural and food-related principles and goals (Principle 1, Principle 2, SDG 14, and SDG 15) are the most prominent. Agriculture and the food industry have been key drivers of the bioeconomy transition in primary and industrial sectors, largely due to their substantial impact on labor productivity (Ronzon et al. 2022). However, aspects, such as collaboration (Principle 10), inclusive and competitive bioeconomic growth (Principle 3), and sustainable water management (SDG 6), gain greater prominence at the regional scale. A case study on Ireland illustrates the challenges of translating the EU’s ambitious bioeconomy goals into national strategies. Key gaps emerge in governance, partnership models, and sustainability approaches, particularly in addressing socio-economic dimensions, highlighting a significant misalignment between policy ambitions and implementation (Faulkner et al. 2024b). Leveraging their comparative advantages, countries like Norway focus on the blue bioeconomy and Finland on the forest-based bioeconomy, aligning with EU goals while prioritizing sectors most relevant to their economies (Gardossi et al. 2023).

Third, bioeconomy strategies strongly emphasize environmental sustainability but largely neglect social dimensions, equity, and collaboration. Most strategies fail to integrate principles of a just, equitable, and fair transition and social equity, focusing instead on economic and environmental aspects, which corresponds with the wider bioeconomy literature foci (Virgolino and Holden 2025). SDG 12, promoting responsible consumption and production, is the most frequently referenced, particularly at the regional level. Research highlights circularity’s role in eco-innovation, resource recovery, and circular-, green entrepreneurship-, and AI-driven business models (Alka et al. 2024) while emphasizing the need for non-toxic, sustainable production processes to protect water, air, and soil (Schröder and Barrie 2024). Therefore, countries link their bioeconomy strategies closely to circular and green economy concepts, yet the sustainability impact remains controversial (Nifatova et al. 2024). Two dominant bioeconomy visions prevail predominantly used in the strategies: a pro-economic growth vision focused on green growth and technological fixes, and a pro-planetary limits vision that challenges growth (Ramcilovic-Suominen et al. 2022). Neither fosters systemic transformation, as pro-growth strategies ultimately reinforce business-as-usual, while planetary-boundary approaches fail to address deeper socio-environmental injustices (Leipold 2021; Holmgren et al. 2022; Ramcilovic-Suominen et al. 2022). Consequently, bioeconomy strategies remain limited in their capacity to drive systemic change (Eversberg et al. 2023). A bioeconomy with sustainability and circularity at its heart requires considering trade-offs and solution approaches beyond the current bias toward green growth or planetary boundaries (Starke et al. 2023). To foster true transformation, policymakers, researchers, and other practitioners need to work toward integrating more evidence on synergies and trade-offs.

Fourth, bioeconomy strategies largely overlook potential unintended consequences for sustainability. They tend to emphasize opportunities while underestimating risks, leading to a gap between data-driven evidence and policy discourse. Challenges, when mentioned, are often framed around innovation deficits, knowledge gaps, and investment needs rather than the broader impacts of the bioeconomy transition. If trade-offs occur, growth-oriented bioeconomy considers them a problem of the future or solvable through technical innovation and improved management (Fleischmann et al. 2024). Of 23 national bioeconomy roadmaps analyzed in 2024, only 6 include concrete, time-dependent actions, governance structures, monitoring, and financing mechanisms (Yang et al. 2024). Despite frequent references to the bioeconomy’s transformative potential, without further measures focusing on the circular use of biomass, its actual contribution to sustainability progress remains limited (Többen et al. 2024; Warchold and Pradhan 2025). A lack of a unified global framework for governing bioeconomies further hampers decision-making regarding international sustainability effects (Yang et al. 2024). Circular economy roadmaps face similar shortcomings, with most failing to account for potential unintended consequences, global value chain dependencies, just transition principles, and adequate financial resources (Schröder and Barrie 2024). Developing a competitive bioeconomy requires policymakers to adopt integrative, multi-level strategies that simultaneously address social, economic, ecological, and circularity concerns, while explicitly recognizing risks and trade-offs as central elements of bioeconomy governance (Ahenkora 2025).

While this study offers valuable insights into the bioeconomy’s role in sustainability transitions, several limitations must be acknowledged. Within our text analysis, computational biases may arise from enumerations, complex visualizations, and multi-column tables and pages, potentially causing biases in keyword mapping. Despite manual verification, some instances may have been overlooked, where authors’ bias could influence interpretations. For example, the Spanish bioeconomy strategy frequently references food loss and waste, with terms like “food”, “loss”, and “waste” appearing multiple times, up to 12 occurrences on a single page, potentially overstating their relative importance. Additionally, the analysis is constrained by the predefined word list of keywords and sentiments, as the presence of positive or negative terms does not necessarily indicate an actual sentiment. To mitigate this, we conducted a thorough manual review of the mapped text. For example, while several bioeconomy strategies include educational, training, and skill development components, directly mapping these to specific SDG 4 targets is challenging. Consequently, the bioeconomy’s impact on some SDGs, though potentially (in)significant, may be indirect and difficult to identify with our method. However, the extent to which the frequency of SDGs mentioned in strategies reflects their urgency for development remains uncertain. Countries that have advanced a goal may focus less on opportunities and more on stability or trade-offs. The sentiment analysis further captures framing, not the depth of engagement. Understanding the potential drivers of change within the bioeconomy is, therefore, essential to ensure a balanced approach that accounts for both opportunities and trade-offs. Further, the frequency of bioeconomy principles and SDGs mentioned in the strategies does not necessarily reflect their consideration while implementing them. The risk of transitioning toward an unsustainable bioeconomy remains, even if principles and SDGs are included in the strategies for precisely this purpose. Our analysis, therefore, interprets opportunities and challenges as an initial reflection of how strategies position the bioeconomy in relation to the SDGs, but it does not yet capture the depth or quality of this engagement.

The discrepancies between the strategies indicate that European countries, as well as other non-European nations, should promote a common understanding of bioeconomy objectives and strategies. Bioeconomy research policy and governance will need to be more strongly focused on achieving international goals, such as the SDGs, and demonstrate the contribution the bioeconomy can make in this context (Schütte 2018). Countries should revise their national plans and refrain from mainstreaming some goals at the expense of others. To increase the importance of the bioeconomy as an effective global tool for achieving the SDGs, it is essential to approach them holistically, considering potential synergies and trade-offs. Although bioeconomy strategies vary significantly in scope and level of detail, they can provide a greater understanding of how the bioeconomy is currently developing in Europe and how it is likely to develop in the future. The SDGs must, therefore, be effectively translated between global, national, and regional aspirations to succeed. Aligning sustainability agendas—such as the bioeconomy—with the SDGs helps guide policies toward long-term sustainable development while ensuring an integrated vision. This alignment ensures that strategies transcend isolated policy actions, fostering systemic change while mitigating trade-offs and maximizing synergies.

## Conclusion

The bioeconomy is increasingly seen as a key element of national development strategies, particularly in Europe, where it is viewed as a means of achieving sustainable economic growth and meeting the objectives of the 2030 Agenda. As such, several countries have developed national and regional bioeconomy strategies to guide their policies and investments. Our analysis of those strategies across scales shows that they are closely aligned with overarching sustainability agendas such as the European Green Deal and the Paris Agreement, with particular emphasis on climate action (Principle 2, SDG 13), forest conservation (Principle 2, SDG 15), and circular economy principles (Principle 5, SDG 12). While strategies strongly emphasize environmental sustainability, they largely neglect social SDGs, equity, and collaboration, with few references to a just and fair transition (Principles 4, 8, 9, & 10). Narratives often state that the bioeconomy offers a realistic opportunity to reconcile economic growth with sustainability and environmental responsibility. However, opportunities and potential synergies are often overstated, while challenges due to trade-offs are frequently neglected, especially by the political community formulating the development strategies.

The 2030 Agenda, with its 17 SDGs, together with the FAO’s framework of ‘*Aspirational Principles and Criteria for a Sustainable Bioeconomy*’, but also the European Green Deal or the European Strategy for Biodiversity, provide a coherent basis for shaping more comprehensive bioeconomy strategies. Aligning future strategies more closely with these agendas can help move beyond rhetorical aspirations, strengthen the recognition of risks and trade-offs, and contribute to more cohesive and impactful acceleration of sustainable development.

## Supplementary Information

Below is the link to the electronic supplementary material.

Supplementary file 1 (ZIP 1650 kb)

## Data Availability

All data supporting the findings of this study are provided in the Supplementary Information.
