# Supplementary material for: European bioeconomy strategies could better integrate sustainability agendas
Source: Sustain Sci. 2025 Oct 13;21(1):77–94. doi: 10.1007/s11625-025-01752-1 (PMC12819565; doi:10.1007/s11625-025-01752-1)
Supplement: Supplementary file 1 — (ZIP 1650 kb) [file 11625_2025_1752_MOESM1_ESM.zip › Supplementary file.pdf]

Supporting data file

Data S1: Search query data based of bioeconomy criteria

Search query data based on the description of bioeconomy criteria.

Data S2: Search query data on SDG targets

Search query data on SDG targets adopted from Pradhan et al. (2024) by adding additional synonyms to capture a broader range of occurrences.

Data S3: SDG sentiment

Overview of sentiments on bioeconomy opportunities and challenges in regards to SGD targets.

Supporting figures

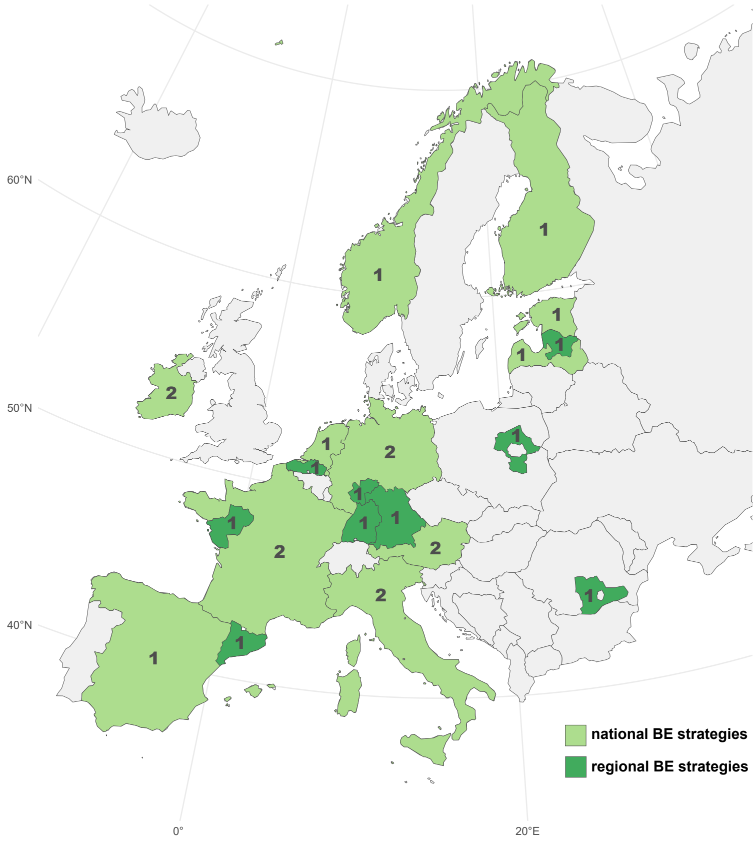

Fig. S1 European Bioeconomy (BE) Strategies: The map shows the national and regional distribution of the 25 strategies included in the analysis, while only strategies in English were considered. The numbers indicate the frequency of strategies within each geographical location. Furthermore, four strategies at the EU level are included.

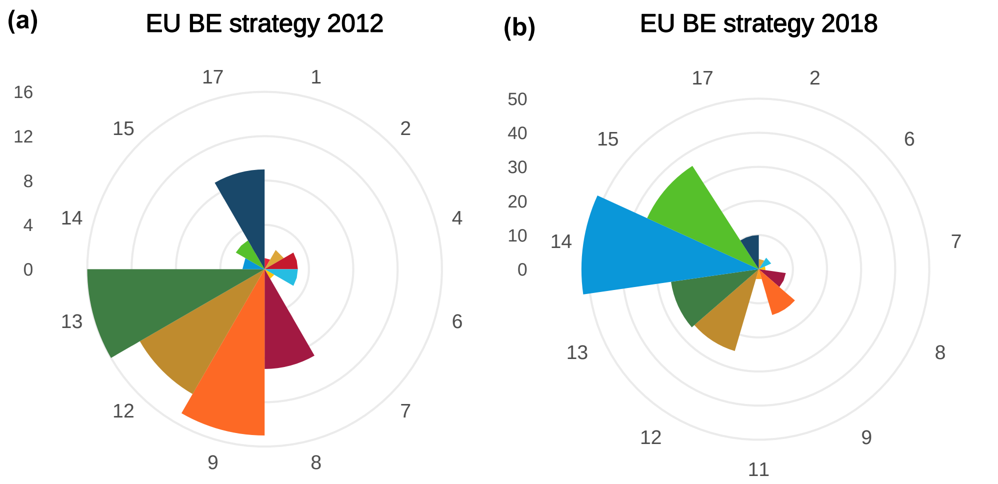

Fig. S2 Frequency of Sustainable Development Goals (SDGs) mapped for 2012 (a) and 2018 (b) Bioeconomy (BE) strategies published by the European Union (EU).

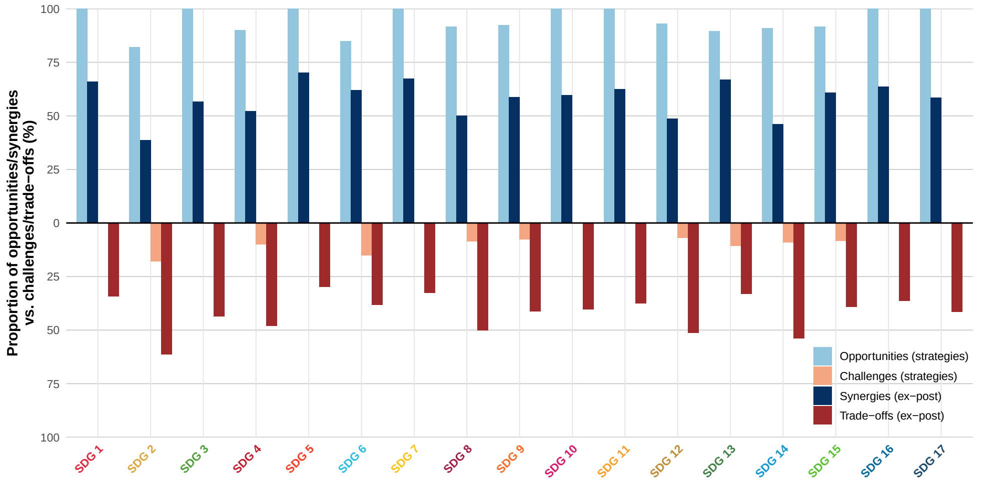

Fig. S3 Bioeconomy (BE) opportunities and challenges in achieving the Sustainable Development Goals (SDGs): as identified in BE strategy documents, and as ex-post evaluations of the BE's positive (synergies) and negative (trade-offs) impacts on SDGs. Opportunities and synergies are represented in blue, while challenges and trade-offs are shown in red. Each bar corresponds to an individual SDG.

Supporting tables

Table S1: Overview of the Food and Agriculture Organization (FAO) and the International Sustainable Bioeconomy Working Group (ISBWG) agreed on a set of "Aspirational Principles for a Sustainable Bioeconomy" (Bracco et al., 2019): The set consists of ten principles (P) and 24 criteria (C) building a framework for a sustainable bioeconomy.

P	Label	Description	C	Description
**P1**	food and nutrition secure	sustainable bioeconomy development should support food security and nutrition at all levels	1.1	food security and nutrition are supported
			1.2	sustainable intensification of biomass production is promoted
			1.3	adequate land right and right to other natural resources are guaranteed
			1.4	food safety, disease prevention and human health are ensured
**P2**	productive and regenerative	sustainable bioeconomy should ensure that national resources are conserved, protected and enhanced	2.1	biodiversity conservation is ensured
			2.2	climate change mitigation and adaption are pursued
			2.3	water quality and quantity are maintained, and, in as much as possible, enhanced
			2.4	the degradation of land, soil, forests, and marine environments is prevented, stopped or reversed
**P3**	competitive and inclusive	sustainable bioeconomy should support competitive and inclusive economic growth	3.1	economic development is fostered
			3.2	inclusive economic growth is strengthened
			3.3	resilience of the rural and urban economic is enhanced
**P4**	resilient and fair	sustainable bioeconomy should support competitive and inclusive economic growth	4.1	the sustainability of urban centres is enhanced
			4.2	resilience of biomass producers, rural communities and ecosystems is developed and/or strengthened
**P5**	efficient and circular	sustainable bioeconomy should rely on improved efficiency in the use of resources and biomass	5.1	resource efficiency, waste prevention and waste re-use along the whole bioeconomy value chain is improved
			5.2	food loss and waste is minimized and, when unavoidable, its biomass is reused or recycled
**P6**	well-governed and transparent	responsible and effective governance mechanisms should underpin sustainable bioeconomy	6.1	policies, regulations and institutional set up relevant to bioeconomy sectors are adequately harmonized

P	Label	Description	C	Description
			6.2	inclusive consultation processes and engagements of all relevant sectors of society are adequate and based on transparent sharing of information
			6.3	appropriate risk assessment and management, monitoring and accountability systems are put in place and implemented
**P7**	innovative	sustainable bioeconomy should make good use of existing relevant knowledge, proven sound technologies and good practices and, where appropriate, promote research and innovations	7.1	existing knowledge is adequately valued and proven sound technologies are fostered
			7.2	knowledge generation and innovation are promoted
**P8**	equitable	sustainable bioeconomy should use and promote sustainable trade and market practices	8.1	local economies are not be hampered but rather harnessed by the trade of raw and processed biomass, and related technologies
**P9**	responsible	sustainable bioeconomy should address societal needs and encourage sustainable consumption	9.1	consumption patterns of bioeconomy goods match sustainable supply levels of biomass
			9.2	demand and supply-side market mechanism and policy coherence between supply and demand of food and non-food goods are enhances
**P10**	collaborative	sustainable bioeconomy should promote cooperation, collaboration and sharing between interested and concerned stakeholders in all relevant domains and at all relevant levels	10.1	cooperation, collaboration and sharing of resource, skills and technologies are enhanced when and where appropriate
